# Economic burden in the management of transfusion-dependent thalassaemia patients in Malaysia from a societal perspective

**DOI:** 10.1186/s13023-021-01791-8

**Published:** 2021-04-07

**Authors:** Asrul Akmal Shafie, Jacqueline Hui Yi Wong, Hishamshah Mohd Ibrahim, Noor Syahireen Mohammed, Irwinder Kaur Chhabra

**Affiliations:** 1 Discipline of Social and Administrative Pharmacy, School of Pharmaceutical Sciences, Universiti Sains Malaysia, 11800 Gelugor, Pulau Pinang Malaysia; 2 Pharmacy Department, Hospital Kuala Lumpur, Ministry of Health Malaysia, Kuala Lumpur, Malaysia; 3 Clinical Research Centre, Hospital Sultanah Bahiyah, Ministry of Health Malaysia, Kedah Darul Aman, Alor Setar, Malaysia; 4 Division of Research and Technical Support, Ministry of Health Malaysia, Putrajaya, Malaysia

**Keywords:** Transfusion-dependent thalassaemia, Lifetime healthcare cost, Patient and family expenditure, Catastrophic healthcare expenditure

## Abstract

**Background:**

Transfusion-dependent thalassaemia (TDT) is a hereditary blood disorder in which blood transfusion is the mainstay treatment to prolong survival and improve quality of life. Patients with this disease require blood transfusion at more than 100 ml/kg annually and iron-chelating therapy (ICT) to prevent iron overload (IOL) complications. There are substantial numbers of TDT patients in Malaysia, but limited data are available regarding the economic burden associated with this disease. The purpose of this study was to determine the lifetime cost of TDT from a societal perspective and identify potential factors increasing patient and family expenditures among thalassaemia populations.

**Methods:**

The total lifetime cost per TDT patient (TC1) is the sum of lifetime healthcare cost (TC2) and lifetime patient and family healthcare expenditure (TC3). TC2 was simulated using the Markov model, taking into account all costs subsidized by the government, and TC3 was estimated through a cross-sectional health survey approach. A survey was performed using a two-stage sampling method in 13 thalassaemia centres covering all regions in Malaysia.

**Results:**

A TDT patient is expected to incur TC2 of USD 561,208. ICT was the main driver of cost and accounted for 56.9% of the total cost followed by blood transfusion cost at 13.1%. TC3 was estimated to be USD 45,458. Therefore, the estimated TC1 of a TDT patient was USD 606,665. Sensitivity analyses showed that if all patients were prescribed oral ICT deferasirox for their lifetime, the total healthcare cost would increase by approximately 65%. Frequency of visits to health facilities for blood transfusion/routine monitoring and patients who were prescribed desferrioxamine were observed to be factors affecting patient and family monthly expenses.

**Conclusion:**

The lifetime cost per TDT patient was USD 606,665, and this result may be useful for national health allocation planning. An estimation of the economic burden will provide additional information to decision makers on implementing prevention interventions to reduce the number of new births and medical service reimbursement.

**Supplementary Information:**

The online version contains supplementary material available at 10.1186/s13023-021-01791-8.

## Introduction

Studies of the economic burden of diseases are useful in health programme financing and planning for a country. Information from these studies allows projection of future cost and priority setting of health programmes. Chronic diseases exert high financial impact on healthcare systems and societies due to the need for long-term care and loss of productivity [1]. However, these can be prevented with early detection or prevention programmes.

Thalassaemia is a hereditary haemoglobin disorder characterized by decreased or absent synthesis of normal globin chains. Patients suffering from this disorder are categorized into two different spectra: transfusion-dependent thalassaemia (TDT) and non-transfusion-dependent thalassaemia (NTDT). TDT patient groups could be diagnosed with either β-thalassaemia major, severe HbE/β-thalassaemia or transfusion-dependent HbH disease traits. These patients would require regular transfusions or more than 100 ml/kg blood per year. Blood transfusions are associated with the risk of iron overload (IOL) at vital organs (heart, liver, endocrine organs), leading to organ damage. Prescribing iron-chelating therapy (ICT) is mandatory to delay IOL complications, which compromise patient quality of life (QOL) and survival. There are three ICTs marketed for clinical use: desferrioxamine (DFO), deferiprone (DFP) and deferasirox (DFX) [2–7].

Over the last three decades, TDT patients were unable to survive up to adulthood. At present, if TDT is treated with appropriate medical and supportive management, the overall survival reported from a Greek study was 65% at the age of 50 years [8]. The largest proportion of β thalassaemia carriers in the population is reported in Maldives (18%), Cyprus (14%), Sardina (10.3%) and Southeast Asia (3–5%) [4, 7]. It was reported that approximately 55 million carriers of thalassaemia live in Southeast Asia [4]. According to the Malaysian Thalassaemia Registry, there are 8681 thalassaemic patients; more than half are TDT patients and are estimated to have 150–300 births of TDT each year [9].

Studies on the economic burden of TDT have been reported in the United Kingdom (UK) [6, 10], United States [11], Italy [12], Iran [13], Thailand [14], Taiwan [15] and India [16]. The lifetime healthcare costs reported from these countries vary between US$ 363,149 and UD$ 720,201. Blood transfusion and ICT costs were found to be the major cost drivers, while a study from Iran claimed that nursing charges were one of the notable expenditures [13]. A literature review on 17 published economic evaluation papers found that direct medical costs represent the largest component of the total cost of TDT, and a significant proportion come from transfusion and ICT costs [17].

The Malaysia public health system is financed by general revenues collected by the federal government. Public sector services are highly subsidized with goods and services free to users or with minimal co-payments. The public health sector is under extreme pressure to control healthcare costs due to population growth and demand [18]. Thalassaemia has become a major public health issue due to its large cost burden, as the majority of the treatment costs were subsidized by the Ministry of Health (MOH) [19].

To our knowledge, no cost-of-illness studies have been conducted on thalassaemia disorder in Malaysia. Cost-of-illness studies are an essential analysis in healthcare planning, and the results provide information to compare the cost of treatment and prevention programmes of the disease if available. A comparison study conducted in Israel reported that the cost of preventing an affected β-thalassaemia newborn was USD 63,660 compared to USD 1.981,380 for treatment of β-thalassaemia major over a lifetime of 50 years [20]. In this study, we aimed to estimate the lifetime costs of TDT patients in Malaysia from a societal perspective. The study results are expected to increase the understanding of the economic implications of managing TDT on the government, patients/families and society. This information is important to assist health policy makers in developing more effective treatment or prevention strategies against the disease.

## Methodology

### Study design

In this study, the economic burden of TDT was estimated from the patient, provider and societal perspectives of Malaysia (refer to Additional file 1: Figure S1).

The total lifetime societal cost of thalassemia (TC1) is estimated by combining the lifetime cost of healthcare providers (TC2) and lifetime patients/family healthcare expenditure (TC3). TC2 is estimated by an incidence-based approach using a Markov model based on information obtained from published literature and clinicians involved in managing TDT in Malaysia.

TC3 was derived through face-to-face interviews in a nationwide cross-sectional study using a set of de novo questionnaires, the TDT Health Utilization Survey (TDTHUS). The TDTHUS was developed specifically for this study and validated (by content and face validity) to ensure that it accurately measures what it claims to measure [21, 22].

The total lifetime cost per TDT patient (TC1) was estimated using Eq. 1.

1 $$\sum Lifetime\,cost\,per\,TDT\,patient\, \left( {TC1} \right) = TC2 + TC3$$

### Estimating lifetime healthcare cost

#### Overview of the model structure

We adopted a state-transition Markov model with a yearly cycle developed by Delea et al. to estimate the lifetime healthcare cost of the TDT population [23]. The structure of the Markov model is shown Additional file 1: Figure S2. All subjects began the simulation in the health state “TDT with no cardiac complications ± endocrine complications”, which represented the time at which the TDT patient started ICT therapy. Survivors could remain in the original state or move to “TDT with cardiac complications ± endocrine complications” in the subsequent year (cycles) or dead state, which is the absorbing state. Patients entering the cohort would be TDT patients prescribed ICT starting from age 2 years and above [2].

The sum of expected costs of all cycles from the model yields the lifetime cost of ICTs, drug administration, routine monitoring and IOL complication management.

#### Model input

This model employed similar transition probability parameters from the existing study, the details of which have been published previously, due to limited local data on IOL complications among TDT patients in Malaysia [23].

Average weight by age (essential to calculate daily dose of ICTs and blood transfusion costs) was derived from weight data collected from TDTHUS. Growth retardation in TDT patients has been recognized and persists despite major treatment advancements in thalassaemia. Children with TDT have a relatively normal growth pattern in the first decade of life. However, the growth velocity will be slowed, and puberty will be delayed due to IOL in endocrine glands. Growth plate fusion is usually delayed until the end of the second decade of life [24, 25]. Therefore, to stratify the age of our TDT population by weight, we categorized all samples into different age range models and selected the best model using simple linear regression (refer to Additional file 1: Table S1).

The annual risk of cardiac death was estimated from an eight-year Greek observational study on patients between 30–40 years [26]. A meta-analysis estimated that DFX would improve compliance compared to DFO by 20.8% (98.1% vs 77.3%) [10]. Base-case values of the model parameters are summarized in Table 1.

**Table 1:** Base-case values of the model parameters

Parameter	Cost (USD)^a^	Cost (MYR)^b^	Value	References
*Iron chelation therapy*				
DFO (per gram)	10.81	44.00		[28]
DFX (per gram)	36.60	149.00		
DFP (per gram)	0.49	2.00		
*Prescribed dosage (mg/kg/day)*				
DFO			47.4	[23]
DFX			24.6	
DFP			75.0	[2]
*Administration (monthly cost)*				
DFO^c^	96.80	393.98		[28]
DFX	0	0		
DFP	0	0		
*Routine laboratory and instrumental monitoring test (monthly cost)*				
TDT patient < 10 years old	55.39	225.42		[29, 35]
TDT patient ≥ 10 years old	106.47	433.33		
*Blood transfusion cost (per transfusion)*				
per millilitre^d^	0.16	0.67		[3, 35]
Blood group and cross matching test	2.7	11		
Blood transfusion equipment	1.14	4.64		
*Compliance (%)*				
DFO-DFP				[10]
DFO arm			77.3	
DFP arm			90.3	
DFX			98.1	
*Cost of IOL complications*				
Cardiac complication, year 1	536.36	2183.00		[31]
Cardiac complication, year 2+				
Diabetes mellitus, year 1	323.10	1315.00		[34]
Diabetes mellitus, year 2+				
Hypogonadism, year 1	175.40	713.88		[28, 35]
Hypogonadism, year 2+	709.69	2888.42		
Hypoparathyroidism, year 1	128.72	523.88		
Hypoparathyroidism, year 2+	580.41	2362.27		
Hypothyroidism, year 1	40.43	164.57		
Hypothyroidism, year 2+	43.59	177.40		
Annual mortality with cardiac disease (%)			0.0062	[26]
Relative risk of death, TDT vs general population			3.9	[23]

^a^Price of 2019

^b^USD 1 = MYR 4.07 (currency conversion index of Feb 2019)

^c^DFO administration includes: battery infusion pump, talaset (infusion port), syringe, WFI and alcohol swab

^d^Unit cost of filtered red cell (FRC). Each pack of FRC = 300 ml (Malaysia Heath Technology Assessment Section Ministry of Health Malaysia, 2017)

##### Resource utilization

The majority of TDT patients in Malaysia are treated in outpatient/daycare settings, and treatment costs are highly subsidized by MOH. All costs are expressed in US dollars (USDs), with the currency exchange ratio for the Malaysia Ringgit (MYR) of MYR 4.07 = 1 US dollar (year 2019 values) [27]. All unit resource costs are summarized in Additional file 1: Table S2.

##### Drug costs

The costs of ICTs were estimated based on the wholesale acquisition cost from the Malaysia drug formulary (year 2019 value) [28]. The unit costs were as follows: DFX = USD$ 0.037 per mg, DFO = USD 0.011 per mg and deferiprone (DFP) = USD 0.0005 per mg.

In local practice, the majority of patients will be initiated with DFX but are switched to the DFO or DFO + DFP regimen after the age of 15. This is done to save cost due to the increasing DFX cost-to-body weight ratio of patients. We verified this by analysing ICT prescriptions in TDTHUS (*p* < 0.001). Therefore, we assumed that TDT patients aged between 2 and 15 years will be treated with DFX and with the DFO or DFO + DFP regimen from age 16 onwards.

##### Administration costs

Among ICTs, only DFO incurs administration costs as it is administered through the subcutaneous route. Both DFX and DFP are orally administered. There is no available publication on the administration cost of DFO. Therefore, it was estimated by interviewing 10 patients. Patients who are treated with DFO require a portable infusion pump to self-administer the drug at home, Thalaset (DFO infusion port), intravenous catheter, alcohol swab, syringe and water for injection (WFI) for DFO administration, and these supplies will be provided by the hospital monthly.

Based on these analyses, the mean per patient administration cost was estimated to be USD 96.80 per month for five weekly doses.

##### Cost of blood and instrumental monitoring

TDT patients need to undergo a series of routine blood investigations and radiography scans to monitor the iron and serum ferritin levels in the body due to regular blood transfusions to prevent or delay IOL complications and monitor disease progression.

The types of investigations required by TDT patients were different for patients below ten years and aged ten years and above according to our local guideline [29]. More investigation will be required for patients aged ten years and above when they reach puberty. All monitoring tests and unit costs are summarized in Additional file 1: Table S3.

##### Cost of IOL complications

IOL complications captured in the model included cardiac complications, diabetes, hypogonadism, hypothyroidism, and hypoparathyroidism.

Cardiac complications often occur during the second decade of life in TDT patients. Over 70% of all deaths were caused by heart failure and arrhythmias. An Italian study found that heart failure (HF) accounts for 66% of all cardiac complications among thalassaemia major patients [30]. Therefore, HF is used to approximate the cost of cardiac complications. The cost of cardiac complications was extracted from a local HF registry-based analysis of a cost-of-illness study, which was USD 536.36 [31].

Iron overload causes fibrosis and fatty replacement of the pancreas, which leads to the development of type 1 or 2 diabetes [32, 33]. In this study, we assumed that patients with diabetes iron overload complications will receive similar treatment to patients who have type 2 diabetes mellitus. The mean cost for patients who attended diabetes ambulatory care services in all MOH facilities in Malaysia was reported to be USD 161.55 half yearly [34].

Treatment costs of hypogonadism, hypoparathyroidism and hypothyroidism were estimated by costing the average resource use in local practice. The clinical pathway and corresponding use of healthcare resources for the respective endocrine complications were first identified by interviewing paediatric and haematology consultants who are involved in the management of TDT in Malaysia. Generally, clinicians follow the current Malaysia guidelines in managing TDT patients [2, 3]. The management of different IOL endocrine complications due to poor compliance with ICTs is summarized in Table S4.

##### Cost of blood transfusion

TDT patients receive 15–20 ml/kg of a regular packed red blood cell transfusion every 3–4 weeks to alleviate the chronic anaemia associated with the disease [2, 3]. Hence, it was assumed that each TDT patient would receive one blood transfusion monthly (12 times annually). See Eq. 2 for the calculation of the lifetime blood transfusion cost per patient, which is expressed as the sum of the product of the transfusion cost multiplied by the probability of patient survival at each cycle (which was simulated by the Markov model). The cost for every resource used was extracted from the Medical Fees Order [35].

2 $$\begin{aligned} & Lifetime\,blood\,transfusion\,cost\,\left( {cTransfusion} \right) \\ & \quad = \sum {\left( {Age\,specific\,weight \times 15\,{\text{mL}}\,{\text{per}}\,{\text{kg}}\,of\,blood \times unit\,cost\,of\,blood\,pack\,and\,infusion\,medical\,items \times 12} \right)} \\ & \quad \quad \times alive\,probablity{\mkern 1mu} at{\mkern 1mu} each{\mkern 1mu} cycle \\ \end{aligned}$$

cTransfusion: lifetime blood transfusion cost per patient.

### Estimating patient and family expenditure and productivity losses

Patient and family expenditures include costs incurred by patients and families to seek treatment, such as out-of-pocket (OOP), healthcare expenses (including transportation) and absenteeism at work.

#### Study design and sampling method

A multi-centre, cross-sectional health survey was conducted in 12 major thalassemia centres covering all regions in Malaysia between May and September 2018. The sample size required in the study was calculated using a respondent-to-item ratio ranging from 5:1 to 15:1 [21]. There were a total of 26 items in the health survey; therefore, the minimum sample size required was 390, taking a ratio of 15 respondents to 1 item.

Respondents were selected using a two-stage sampling method. Malaysia has a total of thirteen states and is divided into five different regions (central, northern, southern, east coast and east Malaysia) based on the geographical location of the individual state. All major thalassaemia centres were identified from each region, followed by the use of a convenience sampling method to recruit study samples. Each centre was required to collect 50 samples.

Inclusion criteria for this survey are as follows: All TDT patients who were at least two years old who required at least eight units of packed red blood cells or more than 100 ml/kg of blood transfused annually [29]. Only patients who are on ICT were recruited. If the patient is less than 18 years old, the caregiver will be interviewed to answer the TDTHUS.

#### Data collection

Patient and family OOP expenditures and productivity losses were identified through interviews using the TDTHUS questionnaire.

Information collected included costs associated with utilization of OTC medications, purchase of prescribed medications from private health facilities, supplementary/herbal or traditional medicines/services, durable and disposable medical devices/items/equipment and daycare admission/outpatient visit charges for routine blood transfusion or follow-up over the last three calendar months.

Transportation costs were defined as the sum of all return journeys to the hospital for TDT-related management, parking fees and toll charges (if any). In the event of missing costs for those travelling by car, the distance travelled (in kilometres, km) was multiplied by the government reimbursement rate [34].

The costs of productivity losses for the caregivers or patient were estimated by multiplying average hourly wage rates by the number of hours spent for TDT-related outpatient and daycare visits [36, 37].

### Analysis

#### Markov model

The model was simulated to calculate lifetime healthcare cost, which includes ICT cost (cICT), cost of IOL complication management (cIOL) and blood and instrumental monitoring costs (cMonitoring). Blood transfusion cost was added separately to estimate the TC2 per patient. From the Markov model simulation, the average life expectancy of our local TDT population is 57.7 years. Hence, the total lifetime healthcare cost per patient was estimated up to age 58.

3 $$\sum lifetime\,healthcare\,cost\,per\,patient\,\left( {TC2} \right) = cICT + cIOL + cMonitoring + cTransfusion\,\left( {from\,equation\,2} \right)$$

TC2: Lifetime healthcare cost per patient, cICT: Lifetime cost of ICT per patient, cIOL: Lifetime cost of IOL complications per patient, cMonitoring: Lifetime cost of routine blood and instrumental monitoring per patient, cTransfusion: Lifetime cost of blood transfusion per patient.

One-way sensitivity analyses were performed on the main cost driver parameters (ICT drug and blood transfusion inputs) to assess their impact on the lifetime healthcare cost.

#### Patient and family expenditure

Costs were expressed as the mean cost of patient and family expenditure from TDTHUS (see Eq. 4).

4 $$Mean\,cost\,of\,patient\,and\,family\,expenditure\,per\,month = \frac{{\sum \left( {cOOP + cTransportation + cProductivity} \right)i}}{n}$$

*i*: costs reported by individual patient, n: total patients (n = 574), cOOP: costs of out-of-pocket expenditure used for TDT management, cTransportation: costs of transportation to health facilities for TDT treatment, cProductivity: productivity losses by patient (18 years and above) or caregiver.

TC3 was calculated by multiplying the mean cost of patient and family expenditure per month by twelve months and the probability of patient survival at each cycle (see Eq. 5).

5 $$TC3 = \left( {mean\,cost\,of\,patient\,and\,family\,expenditure \times 12\,{\text{months}}} \right) \times alive\,probability\,at\,each\,cycle$$

TC3: Lifetime patient and family expenditure for TDT management (per patient).

The distribution of patient and family expenditure cost data was investigated and appropriate statistical methods were applied to address the skewness, excess zeros and heavy right tails [38]. Stepwise multiple regression analysis was employed to analyse factors affecting patient and family expenditure. All analyses were performed using SPSS version 21.0 software.

## Results

### Lifetime cost of TDT patient management

A TDT patient is expected to incur healthcare costs of USD 561,208 from a provider perspective (Table 2). ICT was the main cost driver, accounting for 56.9% of the total cost, followed by blood transfusion cost (13.1%). Lifetime patient and family expenditure is USD 45,458. The total estimated lifetime cost of TDT patients from a societal perspective is USD 606,665.

**Table 2:** Lifetime expected cost per TDT patient at 2018/2019 prices from a societal perspective

Total cost	Item	US Dollar, USD ($)	Malaysia Ringgit,(MYR)	Proportion of total (%)
TC2	ICT drug	345,251.68	1,405,174.32	56.9
	Blood transfusion	79,407.41	323,188.15	13.1
	Routine laboratory and instrumental monitoring test	49,984.71	203,437.75	8.2
	IOL Complications	48,838.38	198,772.20	8.1
	DFO administration	37,725.72	153,543.67	6.2
TC3		45,457.57	185,012.31	7.5
TC1 (TDT patient lifetime cost)		606,665.46	2,469,128.41	100.0

**Fig. 1:**
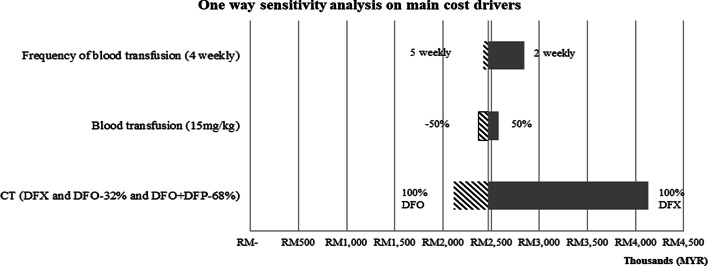
Sensitivity analyses. This figure shows how the cost-of-illness (year 2019 values) changes as blood transfusion and proportion of ICTs parameters changed while holding all constant. The base-case value for each parameter was presented at vertical axis. The base-case estimate of COI is between MYR 2, 000 – 3,000 in thousands. The values at the side of the bars represent the new input values for the respective parameter

Sensitivity analyses showed that if all patients were prescribed DFX for their lifetime, the total healthcare cost would increase by approximately 65% (Fig. 1). The cost of TDT would vary by less than 5% from the base-case value with changes in the volume and frequency of the blood transfusion input parameter.

### Patient/family healthcare expenditure

A total of 574 patients were recruited. The mean age was 17.3 years (SD = 10.6), with the majority coming from Malay ethnicity (Table 3). Twenty-seven percent of patients aged above 18 were unemployed.

**Table 3:** Patient (a) socio-demographic and (b) clinical characteristic

	Frequency (%)
**(a) Description of socio-demographic variables (N = 574)**	
*Region, n (%)*	
Central	160 (27.9)
Southern	80 (13.9)
Northern	72 (12.5)
East Coast	156 (27.2)
East Malaysia	106 (18.5)
*Female patient, n (%)*	317 (55.2)
*Age*	
Mean (SD)	17.3 (10.6)
Median (IQR)	15 (15)
*Age group of patient, n (%)*	
Less than 18 years old	347 (60.5)
18 years old and above	227 (39.5)
*Weight (kg)*	
Mean (SD)	36.8 (16.1)
*Ethnic, n (%)*	
Malay	391 (68.1)
Chinese	95 (16.6)
Indian	3 (0.5)
KadazanDusun	59 (10.3)
Bajau	9 (1.6)
Other-Sabahan native	2 (0.3)
Others	15 (2.6)
*Working/schooling status at enrolment, n (%)*	
Less than 18 years old (n = 347)	
Not schooling	35 (10.1)
Student	310 (89.3)
Not recorded	2 (0.6)
18 years old and above (n = 227)	
Student	36 (15.9)
Adult—working	130 (57.3)
Adult—not working	61 (26.9)
**(b) Description of clinical characteristic**	
*Age at First transfusion as TDT*	
Mode (Min, Max)	1 (0, 50)
*Years of transfusion*	
Mean, SD	13.4 (8.9)
*Presence of IOL complications, n (%)*	
Yes	255 (44.6)
No	319 (55.4)
*Types of current ICT, n (%)*	
Desferrioxamine(DFO)	67 (11.7)
Deferrasirox (DFX)	238 (41.5)
Deferiprone (DFP)	70 (12.2)
DFO + DFP	154 (26.8)
DFO + DFX	32 (5.6)
DFX + DFP	12 (2.1)
DFO + DFP + DFX	1 (0.2)

The majority of patients were transfused at the age of one, with a 13.4-year mean transfusion period (SD = 8.9). DFX (41.5%) was reported to be the highest ICT prescribed, followed by the dual ICT regimen (DFO + DFP) (26.8%). The monthly estimated mean total patient and family resource use is USD65.56 (SD = 283.02). Productivity losses contributed the highest fraction (35.5%), followed by OOP healthcare expenditures (33.5%) and transportation costs (31.1%).

Total patient and family expenditures were used to explore the factors affecting the cost. TC3 was transformed into natural log form to meet the assumption of a normal distribution to proceed with a multiple linear regression model [38–40]. The fitted model can predict 48% of the variability of the cost (adjusted R^2^ = 0.485). The ability to spend among patients and families was driven by income earnings. Frequency to health facilities and patients who were prescribed DFO were found to be factors affecting monthly patient and family expenses. Patient and family expenses are expected to increase by USD 87 with each extra visit to the thalassaemia centre. An additional year of blood transfusion will increase the cost by 1% yearly (refer to Table 4).

**Table 4:** Fitted explanatory model of patient/family expenditure (natural log form of total patient and family expenditure, MYR)

Variables	Unstandardized coefficient		*t*	*Sig*
	*B*	*Standard error*		
Constant	6.654	0.096	69.011	< 0.001
Income category	0.272	0.020	13.264	< 0.001
No. of hospital visits per month	0.294	0.031	9.511	< 0.001
Regime with DFO	0.262	0.065	4.027	< 0.001
Years of blood transfusion	0.009	0.003	2.708	0.007

MLR*: (F (4, 300) = 72.67, *p* < 0.001) with an R^2^ of 0.492 (adjusted R^2^ = 0.485)

*Multiple linear regressions

**Fig. 2:**
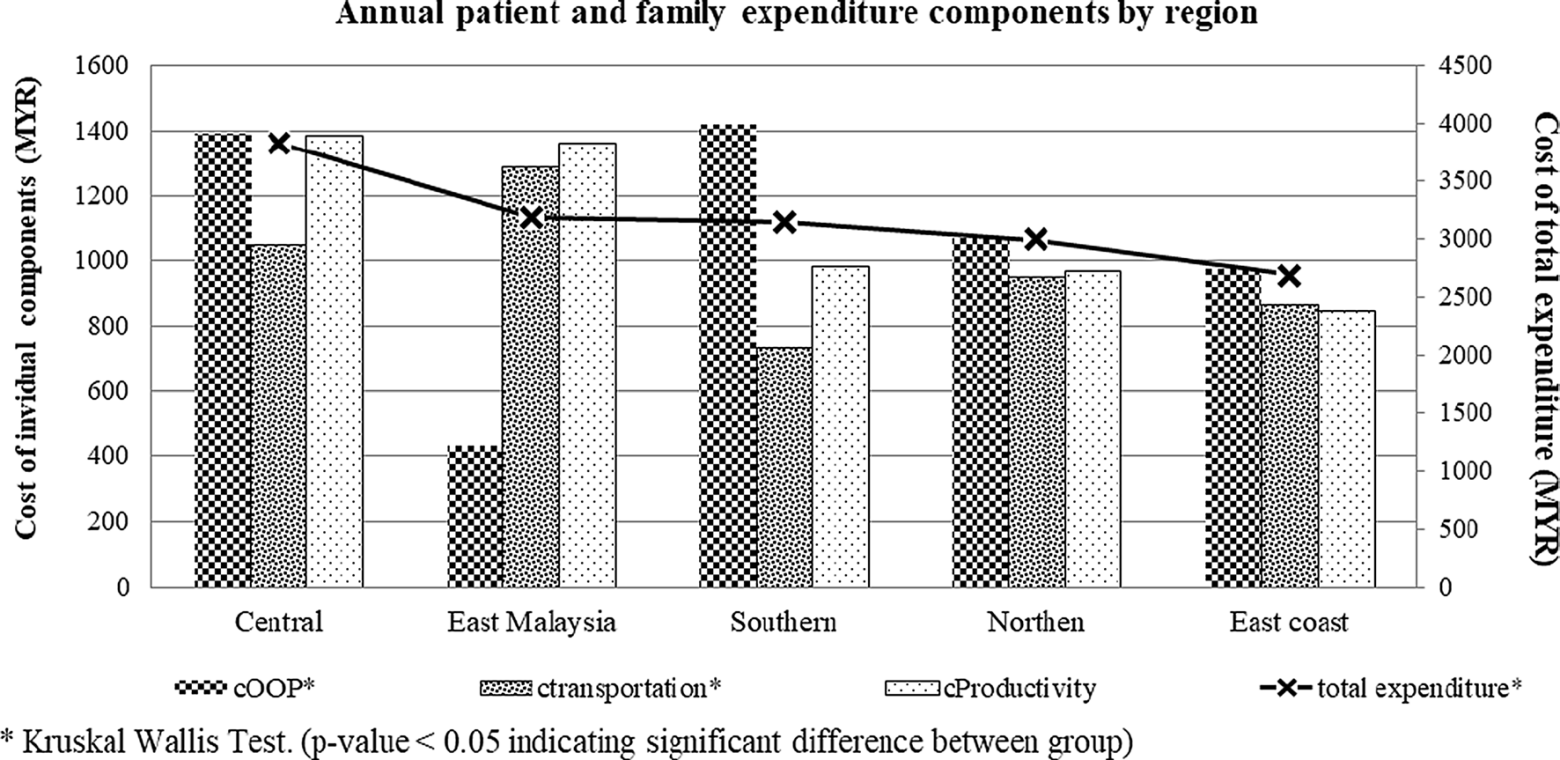
Annual total and individual cost components of patient and family expenditure by region (MYR)

Figure 2 illustrates the yearly total and individual cost components in patient and family expenditures among regions. The central region has the highest expenditure, followed by eastern Malaysia. TDT patients from eastern Malaysia have the highest spending on transportation and are reported to have the lowest cOOP. Central and eastern Malaysia had similar losses in productivity; however, the mean hours spent in the hospital per month was reported to be 11.7 (SD 7.1) vs 22.1 (9.3) hours. There was a significant difference in cOOP, cTransportation and yearly cost of patient and family expenditure (*p* < 0.05) among the five regions in Malaysia.

## Discussion

Although many published studies have reported that thalassaemia disorders, especially transfusion-dependent disorders, impose considerable cost burdens on societies and the healthcare system, there is still inadequate information on their impact on healthcare expenditure. The Malaysia healthcare system is publicly funded; hence, scarce resources limit the feasibility of meeting all patient requirements. This study is the first to estimate the lifetime cost of thalassaemia populations in Malaysia with analysis considering international and Malaysia TDT clinical guidelines and the proportion of patients using common ICT regimens. The estimate of lifetime cost derived in this paper is intended to serve as a national economic burden indicator to care for TDT patients and develop effective strategies to reduce the thalassaemia birth rate.

In this study, the total estimated lifetime cost of managing TDT patients was combined with epidemiological burden data (prevalence) to calculate its national economic burden. The annual cost to manage a TDT patient is substantial at USD 10,499 from a societal perspective. Compared to the neighbouring country of Thailand, the annual average cost of treatment from a societal perspective was found to be approximately USD 950 (at 2005 prices) [14]. A recent Indian study showed that the treatment of TDT was estimated to be USD 1135 annually [41]. National health insurance in Taiwan has reported that the undiscounted lifetime cost in treating TDT patients undergoing conventional therapy was USD 363,149, and our study estimated that the lifetime cost of a TDT patient is USD 561, 208 [15].

The differences in healthcare costs between countries greatly depend on local TDT clinical treatment guidelines and IOL complication treatment pathways as well as the healthcare financing system. If a country does not practice universal healthcare coverage, the cost to be borne by patients and families might be greater [42]. The huge difference in annual cost reported between this study and Thailand TDT management is because in Malaysia, all TDT patients can access healthcare facilities to receive regular blood transfusion, and ICT is provided and subsidized by the Ministry of Health. Younger patients will be prescribed oral ICT and deferasirox (higher in cost), which is convenient to administer to improve compliance. In Thailand, it was reported that some patients are under treatment by international guidelines, and not all patients receive adequate iron chelation. On the other hand, in India, the annual cost is almost the same as that reported in Thailand, and most of the treatment costs were incurred by patients and families or non-government organizations (NGOs).

From an economic perspective, TDT is currently an expensive condition to treat and utilized approximately 1% of the MOH healthcare budget in 2018. The cost of treatment will continue to increase as the survival of TDT patients has moved from lethal at childhood to a large proportion of patients surviving to their 50 s, which is also consistent with the survival probability simulated by the model [4, 15, 43]. The present study revealed that Malaysia has a high economic burden in treating TDT from the provider perspective compared to other Southeast Asian countries, which was attributed to the majority of our patients receiving regular blood transfusions and free accessibility to ICT [4].

In addition to the high magnitude of the economic burden impact on providers, the cost incurred by patients and families may lead to catastrophic health expenditures. Capturing studies over the past 5 years in India, it was reported that a family with a TDT patient spends 38.8% of their annual income on TDT treatment, while in Sri Lanka, the mean household expenditure was approximately USD 206 per year for the care of affected children, and 26.5% of the families experienced catastrophic levels of healthcare expenditure [41, 44]. In Malaysia, families with one TDT child on average spent USD 678 annually (refer to Additional file 1: Table S4), which is approximately 3.3% of the national monthly household income and 5.3% of monthly household income in rural areas [45]. Some of the affected families may have more than one thalassaemic child and hence an increased financial burden consequence that reduces domestic savings and means compromising other social needs and wants of the family. TDT adults (18 years and above), on the other hand, have reported higher expenditures of USD 954 annually compared to TDT children with higher cOOP healthcare expenditures.

In this study, parent or caregiver productivity losses were measured for patients less than 18 years old because TDT is a hereditary disease that requires patients to receive treatment as early as 6 months of age. One of the parents is expected to accompany the patient for regular blood transfusions and frequent follow-ups at the thalassaemia centre. Consequently, this phenomenon will compromise the household income earning ability because only one parent is able to work or must accept pay cuts due to frequent absences at work. However, when compared to parents/caregivers, adult patients were reported to have lower productivity losses despite having to spend more hours in the hospital. (refer to Additional file 1: Table S4). This can be explained by adult TDT patients having poorer earning capacity than healthy caregivers. During the interview, adult patients explained having a salary exploited by employers due to frequent sick leave and poor performance at work resulting from the disorder.

Disparity in TDT management expenditure by patients and families among five regions in Malaysia was observed in this study. In most of the regions, the ability to spend OOP is positively correlated with household income, except for East Malaysia and the northern region. Even though patients from East Malaysia have the lowest income in the country, they have the highest proportion of patient and family expenditures. On average, a TDT patient or caregiver in East Malaysia spends 33.1% of monthly income on TDT-related treatment or expenditures (see Additional file 1: Table S6). East Malaysia patients were reported to visit the hospital more frequently, approximately three times monthly compared to other regions. Frequent visits to healthcare facilities indirectly increase transportation costs and productivity losses (see Fig. 2). The efficiency of TDT management (especially the blood transfusion system) and accessibility in terms of transportation to thalassaemia centres were the major factors causing disparity phenomena among regions. TDT patients, especially those in East Malaysia, have a rare blood phenotype and hence a limited supply of blood from the blood bank. Hence, patients might need to go to the hospital multiple times to receive a sufficient volume of blood transfused to reach the optimal haemoglobin level for better quality of life and to prevent complications.

The lifetime cost of TDT patients estimated from this study was higher than that estimated from other Asian countries, including Thailand [14], Taiwan [15], and Iran [13]; however, comparisons of our results to other countries are difficult due to differences in clinical practices, time horizons and costing methods. Another reason for the higher lifetime cost when compared to neighbouring countries is that the majority of TDT patients receive optimal care from public health facilities, and there is no shortage in terms of ICTs and blood transfusions. Malaysia also complies with international guidelines when treating these patients and is comparable to developed countries such as the UK and US. One of the major strengths of this study is that the estimation of the lifetime cost of TDT was based on national data in terms of unit costs and epidemiologic information. Patient and family expenditure cost components were collected in all thalassaemia centres nationwide. This provides greater generalizability of results. Since this study was conducted nationwide, the results can provide more information on budget planning and implementing prevention strategies by region.

There are several limitations in this paper. Limited published studies or reports were encountered during the literature review on thalassaemia disorder epidemiology data, clinical outcomes and healthcare resource utilization in Malaysia. Apart from that, patient-level disease progression information and age-specific weight for the thalassaemia population were not available. Hence, the model was constructed by incorporating parameter inputs from published studies outside Malaysia and expert opinions from clinicians. As a result, the clinical outcomes and disease progression observed in the model may not accurately reflect our TDT patient cohort.

In terms of healthcare resource utilization, the frequency of resources used was identified through clinical practice guidelines or, if not available, from clinician interviews. It is difficult to standardize treatment pathways and prescribing patterns in clinical practices among paediatricians/haematologists and settings in managing TDT patients. Consequently, the resources used may be different and may not be able to represent all of Malaysia.

Age-specific weight for the thalassaemia population in the model was derived from a single linear regression model (refer to Additional file 1: Table S2). From the regression analysis, the model was able to explain and estimate patient weight by age in the age range of 1–10 years and 11–18 years by 65% and 47%, respectively. However, in patients at the age of 19 and above, the estimated weight can only be explained by 3% with age, as limited growth spurts were expected in this age cohort or weight was affected by other confounding factors, such as diet and disease progression, which were not included in the model. Accuracy in estimating ICTs and blood transfusion costs may be affected, as these costs were substantially associated with patient weight.

Some of the more prevalent complications in older patients were excluded from this analysis. With advancements in TDT management, patients have been reported to survive up to 50 years and above. Hence, planning and budgeting for future services need to be reviewed, as they will probably increase resource use and corresponding costs.

## Conclusion

In conclusion, to the best of our knowledge, this is the first cost-of-illness study of thalassaemia conducted in Malaysia. The result estimated the cost of care to a TDT patient societally. The lifetime cost of TDT patients is estimated to be USD 606,665. Although the majority of the treatment costs were absorbed by the government through universal healthcare coverage, some TDT families still spent a high proportion of their mean monthly income on TDT-related treatment or expenditures, such as transportation costs, over-the-counter medications or disposable medical items for infusion. Hence, in some regions of the country, this scenario could cause a catastrophic financial burden to families with TDT patients.

In view of the substantial economic burden and social and psychological effects of thalassaemia, public awareness of the disease should be addressed at every level. Thalassemia is a monogenic disorder that can be prevented through genetic screening programmes at premarital and antenatal diagnosis. Countries such as Cyprus, Israel, Sardinia and Singapore have proven that screening and prevention programmes are not only effective in reducing the number of thalassaemia newborns; these programmes have been reported to be cost-effective when compared to the cost of providing lifelong supporting treatment to TDT patients [3, 4, 20, 46].

TDT has proven to be a costly disease to be treated; in particular, the main cost drivers are ICTs and blood transfusion costs, which are mandatory to prolong survival and improve the quality of life of TDT patients. Implementing national and effective screening programmes is essential to prevent new cases that will drain our resources, thus helping with economic strengthening. Last, the lifetime treatment cost estimated in this paper is of interest to those involved in the development and provision of screening programmes and to policymakers interested in increasing information on setting priorities regarding this disease.

## Supplementary Information

**Additional file 1.** For economic burden in the management of transfusion-dependent thalassaemia patients in Malaysia from a societal perspective.

## Data Availability

The datasets generated and/or analysed during the current study are not publicly available as it involved third party personal information but are available from the corresponding author on reasonable request.
