# Supplementary material for: Economic burden in the management of transfusion-dependent thalassaemia patients in Malaysia from a societal perspective
Source: Orphanet J Rare Dis. 2021 Apr 7;16:157. doi: 10.1186/s13023-021-01791-8 (PMC8028190; doi:10.1186/s13023-021-01791-8)
Supplement: Supplementary file 1 — Additional file 1. For economic burden in the management of transfusion-dependent thalassaemia patients in Malaysia from a societal perspective. [file 13023_2021_1791_MOESM1_ESM.docx]

**Supplementary material**

Clinical burden

Prevalence of TDT population from Malaysia Thalassaemia Registry (MTR)

=

Economic burden of TDT population

Average treatment cost

Costing from provider’s perspective (estimated from Markov state transition model)

Costing from patient and family perspective (derived from cross-sectional health utilization survey approach)

Figure S1: Conceptual Framework

TDT with **NO** cardiac complication ± endocrine complications (diabetes, hypogonadism, hypoparathyroidism and/or hypothyroidism)

TDT with cardiac complication ± endocrine complications (diabetes, hypogonadism, hypoparathyroidism and/or hypothyroidism)

Dead

Figure S2: Markov model with three health states

Table S1: Age by weight (kg) estimated from 574 samples

| **Age range*** | **β** | | ***t*** | | ***Sig*** | | **R^2^** |
| --- | --- | --- | --- | --- | --- | --- | --- |
|  | **Constant** | **Age** | **Constant** | **Age** | **Constant** | **Age** |  |
| 1-10 years | 6.89 | 1.74 | 9.82 | 13.38 | <0.001 | <0.001 | 0.65 |
| 11-18 years | -4.69 | 2.97 | -1.32 | 12.39 | 0.19 | <0.001 | 0.47 |
| 19 years and above | 44.90 | 0.17 | 20.60 | 2330 | <0.001 | 0.23 | 0.03 |

* Single linear regression was performed for each age range.

| **Age** | **Weight**  **(KG)** | **Age** | **Weight**  **(KG)** | **Age** | **Weight**  **(KG)** | **Age** | **Weight**  **(KG)** |
| --- | --- | --- | --- | --- | --- | --- | --- |
| **1** | 8.63 | **26** | 49.37 | **51** | 53.67 | **76** | 57.97 |
| **2** | 10.37 | **27** | 49.54 | **52** | 53.84 | **77** | 58.14 |
| **3** | 12.11 | **28** | 49.72 | **53** | 54.02 | **78** | 58.32 |
| **4** | 13.85 | **29** | 49.89 | **54** | 54.19 | **79** | 58.49 |
| **5** | 15.59 | **30** | 50.06 | **55** | 54.36 | **80** | 58.66 |
| **6** | 17.33 | **31** | 50.23 | **56** | 54.53 | **81** | 58.83 |
| **7** | 19.07 | **32** | 50.40 | **57** | 54.70 | **82** | 59.00 |
| **8** | 20.81 | **33** | 50.58 | **58** | 54.88 | **83** | 59.18 |
| **9** | 22.55 | **34** | 50.75 | **59** | 55.05 | **84** | 59.35 |
| **10** | 24.29 | **35** | 50.92 | **60** | 55.22 | **85** | 59.52 |
| **11** | 27.98 | **36** | 51.09 | **61** | 55.39 | **86** | 59.69 |
| **12** | 30.95 | **37** | 51.26 | **62** | 55.56 | **87** | 59.86 |
| **13** | 33.92 | **38** | 51.44 | **63** | 55.74 | **88** | 60.04 |
| **14** | 36.89 | **39** | 51.61 | **64** | 55.91 | **89** | 60.21 |
| **15** | 39.86 | **40** | 51.78 | **65** | 56.08 | **90** | 60.38 |
| **16** | 42.83 | **41** | 51.95 | **66** | 56.25 | **91** | 60.55 |
| **17** | 45.80 | **42** | 52.12 | **67** | 56.42 | **92** | 60.72 |
| **18** | 48.77 | **43** | 52.30 | **68** | 56.60 | **93** | 60.90 |
| **19** | 48.17 | **44** | 52.47 | **69** | 56.77 | **94** | 61.07 |
| **20** | 48.34 | **45** | 52.64 | **70** | 56.94 | **95** | 61.24 |
| **21** | 48.51 | **46** | 52.81 | **71** | 57.11 | **96** | 61.41 |
| **22** | 48.68 | **47** | 52.98 | **72** | 57.28 | **97** | 61.58 |
| **23** | 48.86 | **48** | 53.16 | **73** | 57.46 | **98** | 61.76 |
| **24** | 49.03 | **49** | 53.33 | **74** | 57.63 | **99** | 61.93 |
| **25** | 49.20 | **50** | 53.50 | **75** | 57.80 | **100** | 62.10 |

Table S2: Unit resource costs at 2018/19 prices

| **Resource use** | **Unit cost (MYR)** ^a^ |
| --- | --- |
| Mean cost of DFO (mg/kg) | 0.044 |
| Mean cost of DFX (mg/kg) | 0.002 |
| Mean cost of DFP (mg/kg) | 0.15 |
| Monthly cost of DFO administration | 393.98 |
| Unit of red blood cell transfused (cost per mL) | 0.67 |
| Blood transfusion equipment per transfusion | 4.64 |
| Blood group and cross matching laboratory test per transfusion | 11.00 |
| Annual routine monitoring cost for patient less than 10 years | 2705.00 |
| Annual routine monitoring cost for patient more than 10 years | 5200.00 |
| **Management of IOL complications** | |
| Cardiac complications  (annual outpatient and medication cost) | 2183.00 |
| Diabetes complications  (annual outpatient and medication cost) | 1315.00 |
| Hypogonadism, 1^st^ year  Hypogonadism, subsequent year | 713.88  2888.42 |
| Hypoparathyroidism and osteoporosis, 1^st^ year  Hypoparathyroidism and osteoporosis, subsequent year | 523.88  2362.27 |
| Hypothyroidism, 1^st^ year  Hypothyroidism, subsequent year | 164.57  177.40 |

^a.^ 1 US$ = MYR 4.07

Table S3: Costing table for blood and instrumental monitoring test

1. **For TDT patient aged 10 years and below**

| **Test** | **Frequency/**  **year** | **Cost/**  **test (MYR)** ^a^ | **Total cost/**  **year (MYR)** ^a^ |
| --- | --- | --- | --- |
| Full blood count (FBC) | 12 | 40.00 | 480.00 |
| Liver function test (LFT) | 4 | 100.00 | 400.00 |
| Calcium + Phosphate | 4 | 40.00 | 160.00 |
| Serum ferritin | 4 | 20.00 | 80.00 |
| Renal profile | 2 | 80.00 | 160.00 |
| Uric Acid | 2 | 20.00 | 40.00 |
| HIV serology | 2 | 200.00 | 400.00 |
| Hep B serology | 2 | 120.00 | 240.00 |
| Hep C serology | 2 | 210.00 | 420.00 |
| VDRL test | 2 | 30.00 | 60.00 |
| Eye assessment | 1 | 195.00 | 195.00 |
| ENT assessment | 1 | 70.00 | 70.00 |
| **Total monitoring test** | | | **2705.00** |

1. **For TDT patient aged 10 and above**

| **Test** | **Frequency/**  **year** | **Cost/**  **test (MYR)** ^a^ | **Total cost/**  **year (MYR)** ^a^ |
| --- | --- | --- | --- |
| **All routine test in Table 6(a)** |  |  | 2705.00 |
| **Additional test for patient age 10 and above** | | | |
| Modified Glucose Tolerance Test | 2 | 70.00 | 140.00 |
| Thyroid function test | 2 | 65.00 | 130.00 |
| Serum oestradiol/testosterone | 2 | 125.00 | 250.00 |
| Serum cortisol | 2 | 55.00 | 110.00 |
| Echocardiography | 1 | 230.00 | 230.00 |
| MRI T2* of heart and liver | 1 | 1485.00 | 1485.00 |
| Dexa bone scan | 1 | 150.00 | 150.00 |
| **Total monitoring test** | | | **5200.00** |

(Abbreviations: VDRL - Venereal disease research laboratory; ENT – Ear, nose, throat)

^a.^ 1 US$ = MYR 4.07

Table S4: Management of endocrine complications due to iron overload

| **Complications** | **Management of condition** |
| --- | --- |
| Hypogonadism | - Hormonal therapy - Management by an endocrinologist/ paediatrician (outpatient visit) - Routine laboratory and instrumental monitoring |
| Hypoparathyroidism | - Hormonal therapy - Prophylactic vitamin D and calcium tablet - Management by an endocrinologist / haematologist (outpatient visit) - Routine laboratory and instrumental monitoring |
| Hypothyrodism | - Oral replacement of thyroxine - Management by an endocrinologist/ paediatrician/ heamatologist (outpatient visit) - Routine laboratory and instrumental monitoring |

Table S5: Summary of OOP healthcare expenditure, transportation cost and productivity losses incurred by age categories (n=574) in MYR ^a^.

| **Variables** | **Patient less than 18 years old** | | | | **Patient 18 years old and above** | | | |
| --- | --- | --- | --- | --- | --- | --- | --- | --- |
|  | **Patient reporting costs** | | **Total patient (N=347)** | | **Patient reporting costs** | | **Total patient (N=227)** | |
|  | **n** | **Mean (SD)**  **Median (IQR)** | **Mean (SD)** | **Median**  **(IQR)** | **n** | **Mean (SD)**  **Median (IQR)** | **Mean (SD)** | **Median**  **(IQR)** |
| **OOP healthcare expenditure (over 3 months) for TDT management** | | | | | | | | |
| Purchase of OTC medications | 90 | 155.87 (190.32)  120.00 (120.00) | 40.43 (118.31) | 0.00 (18.00) | 47 | 167.57 (174.51)  135.00  (180.00) | 34.70 (104.07) | 0.00 (0.00) |
| Purchase of prescribed medications | 9 | 340.67 (441.05)  210.00  (162.00) | 8.84  (86.24) | 0.00 (0.00) | 11 | 328.91  (441.95)  150.00  (540.00) | 15.94 (116.85) | 0.00 (0.00) |
| Supplement and vitamin | 58 | 284.15 (300.04)  169.50  (291.25) | 47.49 (161.56) | 0.00 (0.00) | 44 | 331.07 (303.37)  201.00 (300.00) | 64.17 (186.32) | 0.00 (0.00) |
| Traditional or alternative treatment/services | 10 | 450.60 (510.57)  240.00 (427.50) | 12.99 (111.71) | 0.00 (0.00) | 13 | 244.62 (184.46)  150.00  (225.00) | 14.01 (71.07) | 0.00 (0.00) |
| Purchase of disposable healthcare equipment | 65 | 154.05 (170.47)  120.00  (144.00) | 28.86 (94.86) | 0.00 (0.00) | 139 | 372.55 (387.74)  240.00 (402.00) | 228.12 (353.40) | 90.00 (300.00) |
| Outpatient and daycare visit charges | 24 | 63.38 (27.78)  60.00 (37.50) | 4.38  (17.63) | 0.00 (0.00) | 155 | 77.15 (71.96)  78.00 (41.00) | 52.68 (69.45) | 54.00 (81.00) |
| Annuitize durable medical equipment | 9 | - | 23.69  (159.67) | - | 38 | - | 189.98  (509.92) | - |
| Total OOP expenditure/month | 347 | - | 47.04  (101.41) | 2.00 (50.00) | 226 | - | 152.97 (191.22) | 88.00  (158.00) |
| **Transportation cost (monthly)** | | | | | | | | |
| **Variables** | **Patient less than 18 years old** | | | | **Patient 18 years old and above** | | | |
|  | **Patient reporting costs** | | **Total patient (N=347)** | | **Patient reporting costs** | | **Total patient (N=227)** | |
|  | **n** | **Mean (SD)** | **Mode**  **(Min, Max)** | **Median**  **(IQR)** | **n** | **Mean (SD)** | **Mode**  **(Min, Max)** | **Median**  **(IQR)** |
| Number of visit to hospital/month  (mode, min-max) | 347 | 1.96 (1.03) | 2 (1,7) | - | 226 | 1.91 (0.63) | 2 (1,4) | - |
| Total transportation cost | 347 | 83.77 (114.97) | - | 49.00 (75.60) | 226 | 80.23 (72.60) | - | 60.00 (72.00) |
| **Productivity losses (monthly)** | | | | | | | | |
| **Variables** | **Patient less than 18 years old** | | | | **Patient 18 years old and above** | | | |
|  | **Patient reporting costs** | | **Total patient (N=347)** | | **Patient reporting costs** | | **Total patient (N=227)** | |
|  | **n** | **Mean (SD)**  **Median (IQR)** | **Mean (SD)** | **Median**  **(IQR)** | **n** | **Mean (SD)**  **Median (IQR)** | **Mean (SD)** | **Median**  **(IQR)** |
| Total hours absence from work (h) | 164 | 13.79 (8.27) | - | - | 141 | 11.29 (7.43) | - | - |
| Monthly Salary, RM |  | 2493.79 (1662.13)  2000.00 (1877.87) | 1173.58  (1692.17) | 0.00  (2000.00) |  | 1897.16 (1402.92)  1400.00 (1000.00) | 1017.11 (1351.65) | 800.00 (1500.00) |
| Total productivity losses |  | 204.26 (183.28)  150.00 (164.75) | 96.07 (162.18) | 0.00 (150.00) |  | 144.64 (203.34)  87.50  (114.38) | 80.98 (174.36) | 29.27 (100.00) |
|  | | | | | | | | |
| **Total patient and family expenditure (monthly)** | 347 | - | 229.95 (257.63) | 150.52  (200.60) | 226 | - | 323.44 (310.23) | 244.20  (239.46) |
| **Total patient and family expenditure (annually)** | 347 | - | 2759.36  (3091.56) | 1806.24 (2407.20) | 226 | - | 3881.33 (3722.76) | 2930.40 (2873.51) |

^a.^ 1 US$ = MYR 4.07

Table S6: Proportion of patient/family expense over monthly income

| **Regions** | **Mean TOTAL patient/family expense/monthly (MYR)^a^** | **Mean monthly income reported by patient/family**  **(MYR)** | **Proportion of TDT patient/family expenditure over average monthly income (%)** |
| --- | --- | --- | --- |
| Central | 310.42 | 1456.82 | 21.3 |
| Southern | 250.40 | 1394.13 | 18.0 |
| Northern | 242.65 | 997.54 | 24.3 |
| East Coast | 214.98 | 1126.35 | 19.1 |
| East Malaysia | 263.05 | 795.52 | 33.1 |
| **Total** | **258.86** | **1178.54** | **22.0** |

^a.^ 1 US$ = MYR 4.07
